# Pantoea phytostimulans sp. nov., a novel strain with plant growth-promoting properties

**DOI:** 10.1099/ijsem.0.006764

**Published:** 2025-04-25

**Authors:** Jonatan C. Campillo-Brocal, María Sánchez-García, Jorge Malo-López-Román, Antonio Bernal-Soro, Antonio Sánchez-Amat

**Affiliations:** 1 Department of Genetics and Microbiology, Faculty of Biology, University of Murcia, Murcia 30100, Spain; 2 Probelte S.A.U., Pol. Ind. la Polvorista, C. Ulea, 22, Molina de Segura 30500, Murcia, Spain

**Keywords:** *Pantoea phytostimulans*, plant growth-promoting rhizobacteria, sorghum

## Abstract

A phosphate-solubilizing bacterial strain, designated C3^T^, was isolated from the rhizosphere of sorghum. The taxonomic classification of this novel isolate was investigated by using a polyphasic approach. Cells of this strain were Gram-stain-negative, facultatively anaerobic and motile short rods with a polar flagellum. Phylogenetic analyses based on the 16S rRNA gene indicated that strain C3^T^ showed high similarity (98.66%) to certain *Pantoea* species, indicating that the isolate belongs to this genus. The genome of strain C3^T^ was 5.05 Mb in size with a G+C content of 51.1 mol%. Whole-genome analysis revealed that the novel strain shared 21.60–27.10% digital DNA–DNA hybridization values and 78.35–83.87% average nucleotide identity with closely related *Pantoea* species; both values below the accepted thresholds for defining a novel species. The predominant cellular fatty acids of strain C3^T^ were C_16:0_, summed feature 3 (C_16:1_ ω6c and/or C_16 :1_ ω7c), summed feature 8 (C_18: 1_ ω7c and/or C_18:1_ ω6c), summed feature 2 (C_12: 0_ aldehyde and/or C_14:0_ 3OH and/or C_16:1_ iso I and/or an unidentified fatty acid of 10.9525 chain length) and C_17:0_ cyclo. Apart from the ability to solubilize phosphates, stain C3^T^ exhibited other *in vitro* plant growth-promoting activities such as K, Ca and Si solubilization, and the production of indole-3-acetic acid (IAA) and siderophores. Several genes related to phosphate solubilization, siderophore biogenesis and IAA, cytokinin and gamma-aminobutyric acid production were identified in the C3^T^ genome. Summarizing, based on phylogenetic, genomic, phenotypic and chemotaxonomic criteria, the isolate represents a novel species of the genus *Pantoea,* for which the name *Pantoea phytostimulans* sp. nov. is proposed. The type strain is C3^T^ (=CECT 31163^T^=NCIMB 15596^T^).

## Introduction

The genus *Pantoea* is a group of Gram-stain-negative, facultative anaerobic, coccoid to rod-shaped gammaproteobacteria, belonging to the family *Erwiniaceae* [1], which was first described by Gavini *et al*. in 1989 [2]. Historically, this taxon has been particularly challenging to classify based on phenotypic traits, as it shares many features with other genera like *Erwinia*, *Enterobacter* and *Pectobacterium* [3]. However, the development of DNA analysis techniques provides nowadays a more robust classification of the genus *Pantoea* [4,5]. As of September 2024, there are 28 validly published *Pantoea* species on the List of Prokaryotic Names with Standing in Nomenclature (LPSN) and, notably, there are 36 additional species, including ‘*Candidatus* Pantoea spp.’, that have not been validly published and therefore hold no official standing (https://lpsn.dsmz.de/genus/pantoea) [6].

As implied by the name of the genus *Pantoea* (‘of all sorts and sources’), this group of bacteria comes from diverse origins, including plants, animals, soil, water environments and even humans [7]. Certain species, such as *Pantoea agglomerans* and *Pantoea dispersa*, have been reported to cause opportunistic infections in humans, particularly in immunocompromised patients [8,9]. Nonetheless, it is worth mentioning that some cases of *Pantoea* infections reported in humans may result from pathogen misidentification [10]. Other species, such as *Pantoea stewartii* and *Pantoea ananatis*, are identified as plant pathogens, causing leaf blight in corn and white spot disease, respectively [11,12]. On the contrary, many strains can play a beneficial role in plant growth. Numerous *Pantoea* isolates exhibit plant growth-promoting (PGP) traits, including macro and micronutrient solubilization, nitrogen fixation, siderophores and phytohormones production [13,16]. Most of these micro-organisms colonize the rhizosphere of their host plants, promoting their growth, and are, therefore, classified as PGP rhizobacteria [17]. The isolation of new PGP bacteria is of great biotechnological interest in agroindustry, as they are expected to contribute to sustainable farming practices by minimizing the use of chemical fertilizers and enhancing soil health while boosting crop yields [18].

*Sorghum bicolor*, a C4 flowering plant in the *Poaceae* family, is highly valued in agriculture, particularly in semiarid regions, due to its strong drought and heat tolerance [19]. It has been proposed that the root microbiota may significantly contribute to the growth of sorghum in these challenging environments, due to different PGP mechanisms [20]. To isolate micro-organisms with such beneficial properties, sorghum roots were sampled, and culturable bacteria were investigated.

In this study, a phosphate-solubilizing strain, named C3^T^, was isolated. We have used a polyphasic taxonomic approach to characterize this new isolate. Analyses based on 16S rRNA gene phylogeny, whole-genome sequencing and phenotypic and chemotaxonomic data suggest that strain C3^T^ represents a novel species within the genus *Pantoea,* for which the strain name *Pantoea phytostimulans* sp. nov. is proposed.

## Isolation and ecology

Strain C3^T^ was isolated from the rhizosphere of sorghum (*S. bicolor*) growing in the southeast of Spain (Jumilla, Murcia; 38° 30′ 41.87″ N 1° 12′ 6.32″ W). Sorghum roots were washed with sterile water to eliminate loosely attached soil and particles. Fine roots (<2 mm) were then excised, macerated in 0.85% sterile saline MiliQ water with a mortar, serially diluted and plated on National Botanical Research Institute’s phosphate growth medium (NBRIP) for screening phosphate-solubilizing micro-organisms [21]. After 72 h of growth at 28 °C, a single bacterial colony with the highest phosphate-solubilized halo was picked and purified to obtain a pure culture by streaking twice on fresh NBRIP plates. This isolate was able to grow in nutrient agar showing yellow-pigmented colonies and was designated strain C3^T^.

## 16S rRNA gene phylogeny

**Fig. 1.**
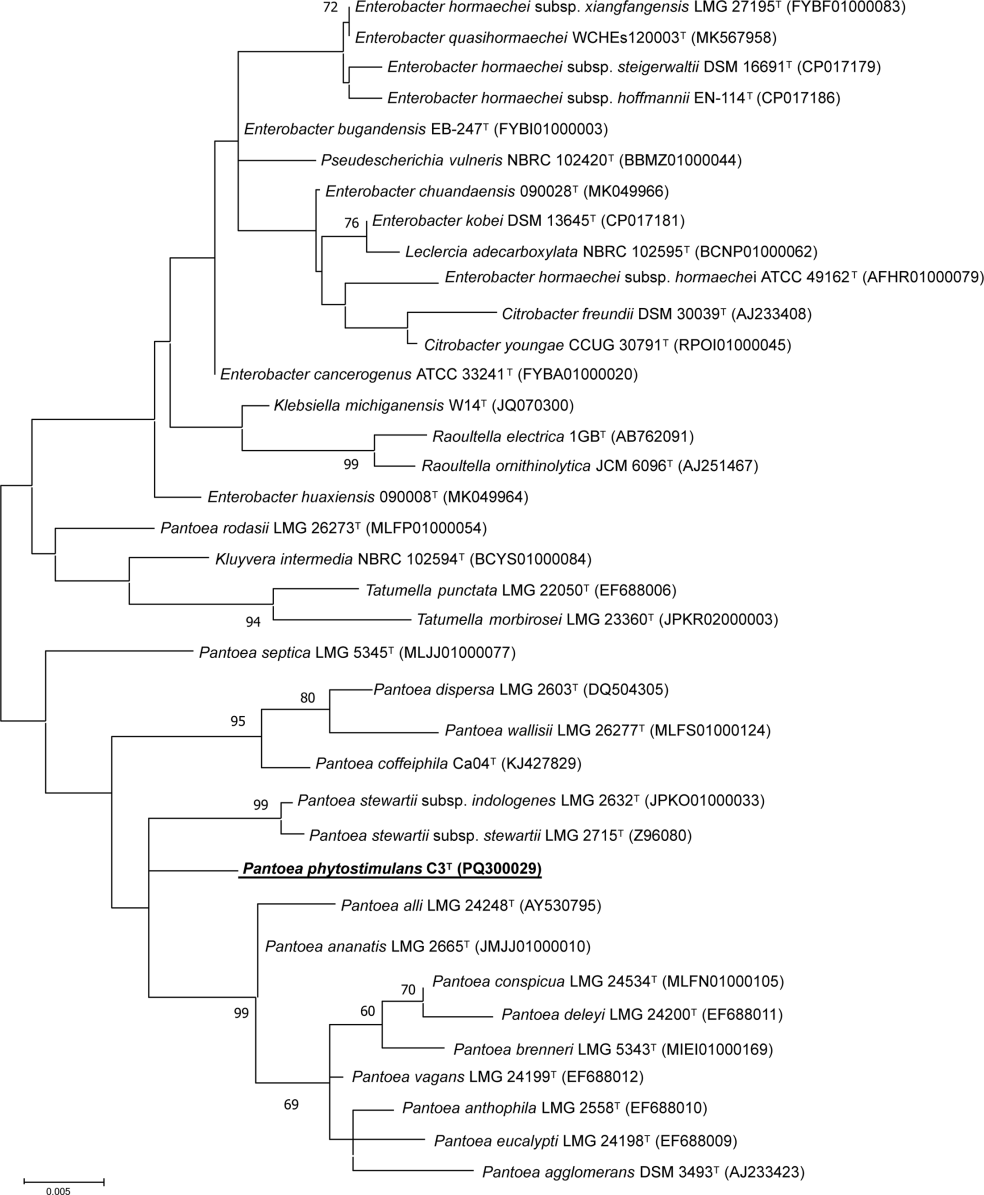
Unrooted maximum-likelihood tree based on 16S rRNA sequences illustrating the closest phylogenetic relatives of *Pantoea phytostimulans* sp. nov. C3T. Bootstrap values, shown as percentages of 1000 replicates, are indicated at the branching points when >60%. Database accession numbers are provided in parentheses. Bar, 0.005 substitutions per nucleotide position.

In a preliminary taxonomic characterization of the isolate, the 16S rRNA gene sequence was analysed. For this, genomic DNA was extracted using the Wizard® Genomic DNA Purification Kit (Promega) according to the manufacturer’s instructions. The 16S rRNA gene was partially amplified by PCR using the universal primers 27F and 1389R [22] and Sanger sequenced in the Área Científica y Técnica de Investigación (ACTI) of the University of Murcia (UMU). The sequences obtained were assembled and edited using BioEdit software (http://bioedit.software.informer.com/). The resulting assembled sequence of 1,268 bp was deposited in GenBank under the accession number PQ300029. The closest known relatives of this 16S rRNA partial gene were identified using the EzBioCloud server (www.ezbiocloud.net) [23]. The highest sequence similarity was 98.66% with *P. ananatis* LMG 2665^T^, *Pantoea stewartii* subsp. *indologenes* LMG 2632^T^ and *Pantoea stewartii* subsp. *stewartii* LMG 2715^T^ and 98.65% with *Pantoea vagans* LMG 24199^T^. These values are slightly below the commonly accepted cut-off of 98.7% used for delineating a novel species [24]. After retrieving the most similar sequences in the EzBioCloud server, they were aligned with strain C3^T^ using clustal w [25], and phylogenetic analysis was conducted using mega program (v11) [26]. Phylogenetic trees were reconstructed using the maximum likelihood (ML) and neighbour-joining (NJ) algorithms [27,28], where genetic distances were calculated using the Kimura two-parameter model and bootstrap values were determined from 1,000 replications [29,30]. The ML-based phylogenetic analysis, shown in Fig. 1, indicates that strain C3^T^ clusters within the genus *Pantoea* but forms its distinct lineage, setting it apart from other species. The phylogenetic tree generated using the NJ method revealed a similar clustering for the close relatives of strain C3^T^ (Fig. S1, available in the online Supplementary Material).

## Genome features and phylogenomics

For genomic sequencing, strain C3^T^ was grown aerobically at 30 °C for 48 h on nutrient broth and DNA was extracted using the Wizard Genomic DNA Purification Kit (Promega). The genome was sequenced by the NextSeq 2000 Illumina platform at the ACTI (UMU) and sent to LongSeq Applications (Murcia, Spain) for sequencing using the Oxford Nanopore GridION device (ONT). Briefly, the bioinformatic pipeline used for building the assembly consisted of Canu for the first assembly [31], Nanopolish for polishing the assembly [32] and Unicycler for improving the assembly with short reads [33]. The assembly was annotated with Prokka [34], and rRNA and tRNA were predicted using Barrnap [35] and tRNAscan [36]. The parameters of the final assembly were 5 contigs with a total genome size of 5.1 Mb, 630× sequencing coverage and 97.2% and 2.5% of estimated completeness and contamination, respectively, based on the CheckM program (v1.2.3) [37]. The genome of C3^T^ included one circular chromosome of 3,989,142 bp and four plasmids presenting plasmid replication *repB* genes, consisting of three megaplasmids, namely pC3_1 (456,038 bp), pC3_2 (391,382 bp) and pC3_3 (218,007 bp), and one short plasmid of 3,609 bp (pC3_4) (Table 1). While different plasmids (including megaplasmids) have been reported in various *Pantoea* species [38,40], those found in strain C3^T^ show no similarity to any previously described within the genus. The genome contained 4,676 protein-coding sequences, 22 rRNA genes and 79 tRNA genes and exhibited a DNA G+C content of 51.5 mol%, which is consistent with the range reported for other species within the genus *Pantoea* (49.7–61.3 mol%) [41]. The whole-genome sequencing data obtained for strain C3^T^ were deposited in GenBank under accession numbers CP173698 for the chromosome and CP173699-CP173702 for the plasmids.

**Table 1.** General genome features for strain C3^T^

	Chromosome	Plasmid				Total
		pC3_1	pC3_2	pC3_3	pC3_4	
Size (bp)	3,989,142	456,038	391,382	218,007	3,609	5,058,178
G+C content (mol%)	51.5	50.1	49.6	48.2	50.9	51.1
Genes	3,782	428	363	202	3	4,778
CDS	3,680	428	363	202	3	4,676
rRNA (5S, 16S, 23S)	22 (8, 7, 7)	0	0	0	0	22
tRNA	79	0	0	0	0	79
tmRNA	1	0	0	0	0	1
GenBank accession	CP173698	CP173699	CP173700	CP173701	CP173702	GCA_046118715.1

CDS, coding sequence.

To further analyse the taxonomic status of strain C3^T^ in the genus *Pantoea*, we performed average nucleotide identity (ANI) calculations, digital DNA–DNA hybridization (dDDH) tests and a whole genome-based phylogenetic tree between strain C3^T^ and the closest related type species. The ANI calculation was performed using the online ANI calculator available on EzBioCloud, which utilizes the OrthoANIu algorithm (https://www.ezbiocloud.net/tools/ani) [42]. The estimated dDDH values were calculated by Genome-to-Genome Distance Calculator 3.0 (https://ggdc.dsmz.de/) using Formula 2 as recommended [43]. The whole genome-based tree was inferred using the Genome blast Distance Phylogeny approach from the Type (Strain) Genome Server (TYGS) (https://tygs.dsmz.de/) [44], under the algorithm ‘coverage’ and distance formula *d_5_* [43].

**Fig. 2.**
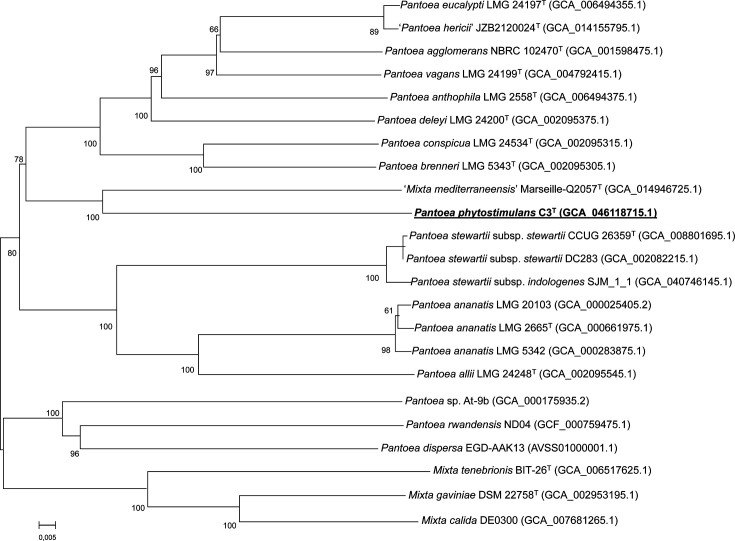
Tree inferred with FastME 2.1.6.1 from GBDP distances calculated from genome sequences of C3T closely related species. The branch lengths are scaled in terms of GBDP distance formula d5. The numbers above branches are GBDP pseudo-bootstrap support values when >60% from 100 replicates. The tree was rooted at the midpoint. Genome accession numbers are given in parentheses. Species names written in quotation marks are not validly published (https://lpsn.dsmz.de). *Mixta calida* DE0300, *Mixta gaviniae* DSM 22758 and *Mixta tenebrionis* BIT-26 were used as outgroups. Bar, 0.005 substitutions per site.

The genome-derived ANI and dDDH values between C3^T^ and its most closely related species were between 78.35–83.87% and 21.60–27.10%, respectively (Table 2). These values are all below the recommended cut-off values for prokaryotic species delineation, i.e. 95–96% for ANI and 70% for dDDH [45]. In addition, the whole genome-based phylogenetic analysis shows that strain C3^T^ forms a separate clade within the genus *Pantoea* (Fig. 2). All these analyses support that strain C3^T^ constitutes a novel species in the genus *Pantoea*.

**Table 2.** The ANI and dDDH values between strain C3^T^ and the most closely related species Species names written in quotation marks are not validly published (https://lpsn.dsmz.de).

Strain	OrthoANIu (%)	dDDH (%)	Accession no.
‘*Mixta mediterraneensis’* Marseille-Q2057^T^	83.87	27.10	GCA_014946725.1
*Pantoea conspicua* LMG 24534^T^	80.73	23.30	GCA_002095315.1
*Pantoea brenneri* LMG 5343^T^	80.63	23.40	GCA_002095305.1
*P.agglomerans* NBRC 102470^T^	80.22	23.10	GCA_001598475.1
*P.vagans* LMG 24199^T^	79.96	23.00	GCA_004792415.1
*Pantoea deleyi* LMG 24200^T^	79.85	22.80	GCA_002095375.1
*Pantoea eucalypti* LMG 24197^T^	79.89	22.70	GCA_006494355.1
*Pantoea anthophila* LMG 2558^T^	79.63	22.70	GCA_006494375.1
‘*Pantoea hericii*’ JZB2120024^T^	79.97	22.60	GCA_014155795.1
*P.stewartii* subsp. *stewartii* DC283	78.66	21.80	GCA_002082215.1
*Pantoea allii* LMG 24248^T^	78.59	21.70	GCA_002095545.1
*P.ananatis* LMG 5342	78.74	21.70	GCA_000283875.1
*P.ananatis* LMG 2665^T^	78.66	21.60	GCA_000661975.1
*P.stewartii* subsp. *stewartii* CCUG 26359^T^	78.70	21.60	GCA_008801695.1
*P.stewartii* subsp. *indologenes* SJM_1_1	78.35	21.60	GCA_040746145.1

Curiously, the strain most closely related to C3^T^ in the tree generated by TYGS is *Mixta mediterraneensis* Marseille-Q2057 [46], which is not a validly published species (https://lpsn.dsmz.de/species/mixta-mediterraneensis). Our taxonomic analysis, which shows that all other validly published *Mixta* strains cluster together in a clade separate from *Pantoea* species, raises questions over the classification of strain Marseille-Q2057 (Fig. 2). In addition, it is worth mentioning that strain C3^T^ shares high ANI and dDDH values (98.46% and 88%, respectively) with the genome of the uncultured bacterium named *Candidatus* Pantoea bituminis EnvD [5]. This isolate was named under the SeqCode, which is not an officially recognized code of nomenclature for the LPSN [47].

## Genes involved in plant growth promotion and secondary metabolites

To gain a preliminary understanding of the PGP capabilities of strain C3^T^, the genome was analysed via the SEED server using the Rapid Annotation Subsystem Technology [48,49]. Genes contributing to plant growth promotion were identified using NCBI blastp with PGP-related proteins from closely related species (https://blast.ncbi.nlm.nih.gov/Blast.cgi). Secondary metabolite-associated biosynthetic gene clusters (BGCs) were determined using antiSMASH (v7.1.0) [50]. Additionally, a comparative genomic analysis was performed with its closest phylogenomic relatives, *M. mediterraneensis* Marseille-Q2057^T^ and *Pantoea conspicua* LMG 24534^T^, to identify differential genetic elements.

Genome mining of strain C3^T^ identified key genes associated with widespread traits among PGP *Pantoea* strains, including phosphate solubilization and the production of siderophores, auxins, cytokinins and gamma-aminobutyric acid (GABA) [13,14, 16]. Regarding phosphate solubilization and transport (Table S1), genes associated with gluconic acid production were detected, including three *gcd* genes encoding glucose dehydrogenase and the *pqq* genes essential for its cofactor synthesis. In addition to gluconate, a major factor in mineral phosphate solubilization, *gad* genes coding for gluconate 2‐dehydrogenase indicate the potential for 2-ketogluconate production, further enhancing this activity [51]. Genes involved in inorganic polyphosphate degradation, including *ppx* (exopolyphosphatase) and *ppa* (inorganic pyrophosphatase), were identified in the C3^T^ genome (Table S1). Additionally, genes encoding extracellular enzymes for organic phosphate solubilization, such as *phoA* (alkaline phosphatase), *appA* (phytase) and *agp* (glucose-1-phosphatase), were present in the genome. The *phn* gene cluster, associated with phosphonate transport and degradation, as well as the *pst* phosphate transport system genes, was also detected, highlighting the potential of C3^T^ for diverse phosphorus acquisition strategies (Table S1). Most of these genes related to phosphate solubilization are present in closely related C3^T^ species, with some exceptions. C3^T^ can be distinguished from *M. mediterraneensis* Marseille-Q2057^T^ and *P. conspicua* LMG 24534^T^, as both lack phytase homologues in their genomes. In addition, strain LMG 24534^T^ also lacks alkaline phosphatase homologues, while Marseille-Q2057^T^ contains an incomplete *phn* gene cluster.

Siderophores are low-molecular-weight compounds with a strong affinity for metal ions, primarily ferric, which can enhance plant growth and development by increasing iron availability [52]. Genome analysis of strain C3^T^ revealed the presence of *ent* and *fep* clusters involved in enterobactin biogenesis and transport (Table S2). The widespread occurrence of these gene clusters across diverse *Pantoea* genomes strongly suggests that enterobactin production is a conserved trait within the genus [53]. Moreover, C3^T^ contains the hydroxamate siderophore transport genes *fhu* (Table S2). Since no gene cluster involved in its synthesis was detected in this study, this suggests that the strain may acquire siderophores produced by other organisms.

Genes encoding phytohormones and other plant-stimulating molecules were also identified in the C3^T^ genome (Table S3). Various genes related to the tryptophan operon (*trpH*, *trpE*, *trpD*, *trpCF*, *trpB*, *trpA*, *trpS* and *trpR*), along with *ipdC* and *aldH* (encoding indolepyruvate decarboxylase and aldehyde dehydrogenase, respectively), suggest that strain C3^T^ produces indole-3-acetic acid (IAA) via the IPyA pathway (Table S3). The presence of *miaA* and *miaB* (encoding tRNA dimethylallyltransferase and tRNA-2-methylthio-N6-dimethylallyladenosine synthase, respectively) and xanthine dehydrogenase genes (*xdhA*, *xdhB* and *xdhC*) indicates that this strain also has the genetic potential for cytokinin production [53]. The production of GABA is also likely in strain C3^T^, as the *puuD* gene (encoding gamma-glutamyl-gamma-aminobutyrate hydrolase), which is involved in GABA biosynthesis, and the *puuR*, *puuE*, *gabT* and *gabD* genes, associated with GABA degradation [53], were also detected (Table S3).

Analysis of the C3^T^ genome using the antiSMASH tool revealed the presence of different BGCs, including the enterobactin cluster (Table S4). Notably, two BGCs were detected associated with the production of pigments, i.e. aryl polyenes and carotenoids. This finding aligns with the observation of yellow-pigmented colonies when the strain is cultured in rich media (Fig. S2). The aryl polyene BGC showed 94% similarity to that in *Xenorhabdus doucetiae*, while the carotenoid BGC displayed 100% identity to the one in *P. ananatis*. These bacterial pigments play crucial roles in cellular defence, acting as antioxidants against reactive oxygen species and as photoprotectants [54,55]. Enterobactin, aryl polyenes and carotenoids BGC are also detected in the closest relatives *M. mediterraneensis* Marseille-Q2057^T^ and *P. conspicua* LMG 24534^T^. However, C3^T^ is unique among these strains in possessing the photobactin BGC, a catechol siderophore typically produced by *Photorhabdus luminescens* [56] (Table S4).

## Physiology and chemotaxonomy

Cell morphology, size and the presence of flagella were examined using phase-contrast microscopy (BH2, Olympus) and transmission electron microscopy (JEM 1011, JEOL). Gram staining was performed following the standard protocol [57]. To determine the optimal growth conditions, we followed the growth of C3^T^ in nutrient agar plates for up to 5 days, at different temperatures (4–40°C, at 4°C intervals), pH levels (4–10, at 0.5 pH unit intervals) and salt concentrations (0–10%, at 1% intervals of NaCl (w/v)). Traditional and standard biochemical tests were used to further characterize the bacterium. Oxidase activity was assessed by the oxidation of 1% (w/v) tetramethyl-p-phenylenediamine, whereas catalase activity was evaluated by the formation of oxygen bubbles in a 3% (v/v) aqueous hydrogen peroxide solution. We also conducted API 10 S and API 50 CH test kits according to the manufacturer’s instructions (bioMérieux, France). To further characterize strain C3^T^ according to its PGP characteristics, we tested different culture media: NBRIP [21], modified Aleksandrov [58], calcite [59] and NBRISSM [60], for, respectively, P, K, Ca and Si solubilization; chrome azurol S agar for siderophore production [61]; IAA production was determined with Salkowski reagent following the protocol in [62]; carboxymethylcellulose agar for cellulase activity [63]; and nutrient agar supplemented with 10% skim milk or with 1% starch for protease (caseinase) or amylase activity, respectively [64].

Strain C3^T^ grown for 72 h on nutrient agar exhibited yellowish-pigmented, moist, convex and circular colonies with a diameter of 1.0–2.5 mm (Fig. S2). Microscopic examination revealed Gram-stain-negative short rod cells, measuring ~0.8–1.2×1.6–2.6 µm in size. The strain was observed to be motile due to the presence of a polar flagellum (Fig. S2). Strain C3^T^ exhibited growth within a temperature range of 4–36 °C (optimum, 28–30 °C), a pH range of 5–9 (optimum, 6.5) and in the presence of 0–7% (w/v) NaCl (optimum, 1%). These characteristics align with the general description of the genus *Pantoea* [41]. Similarly to its closest related species, strain C3^T^ was able to produce acid from d-glucose and d-fructose, but in the fermentation of glycerol, d-maltose, d-lactose, sucrose, l-arabinose, d-ribose and d-mannitol, it differed from at least one of its phylogenomic neighbours. A detailed comparison of differential phenotypic traits between C3^T^ and its closest relatives is outlined in Table 3, while results from biochemical commercial tests can be found in Table S5.

**Table 3.** Phenotypic characteristics of *P. phytostimulans* C3^T^ and its closest neighbours from the phylogenomic analysis Species names written in quotation marks are not validly published (https://lpsn.dsmz.de).

	*P. phytostimulans* C3^T^	‘*M. mediterraneensis*‘ Marseille-Q2057^T^	*P. conspicua*LMG 24534**^T^**	*Pantoea brenneri* LMG 5343**^T^**	*P. agglomerans* NBRC 102470**^T^**	*P. vagans*LMG 24199**^T^**
**Morphology and growth conditions**						
Colony colour	Yellow	Beige	np	np	Yellow	Yellow
Cell shape	Short rod	Rod	np	np	Short rod	Short rod
Cell size (μm)	0.8–1.2×1.6–2.6	0.8×3.8	0.9×1.5–3	0.9×1.5–3	0.5–1×1–3	0.9×1.5–3
Motility	+	−	+	+	+	+
Temperature range (optimum) (°C)	4–36 (28–30)	21–56 (31)	np (28–30)	np (28–30)	np (30)	10–42 (30)
pH range (optimum)	5–9 (6.5)	NP (5–7.5)	np	np	np	4–11 (7–9)
Salinity tolerance (optimum) (NaCl%)	0–7 [1]	np	np	np	np	0–7 (np)
**Acid from**						
Glycerol	w	np	+	−	+	+
d-Maltose	+	−	+	+	+	−
d-Lactose	−	−	+	+	−	w
Sucrose	−	np	+	+	np	+
l-Arabinose	+	−	+	+	+	+
d-Ribose	+	−	+	+	+	+
d-Mannitol	+	−	+	+	+	+
**Genome features**						
G+C content (mol%)	51.1	51.8	55.7	55.4	55.1	55.4
ANI with C3^T^ (%)	100.0	83.87	80.73	80.63	80.22	79.96

All data retrieved from [46,68], except data for *P. phytostimulans* C3T (this study). All strains produced acids from d-glucose, d-fructose and *N*‐acetylglucosamine. +, Positive; w, weak positive; −, negative; np, not provided.

Strain C3^T^ tested negative for cellulase, protease and amylase activities. However, apart from solubilizing inorganic P, strain C3^T^ showed other *in vitro* PGP traits including K, Ca and Si solubilization, as well as the production of siderophores and IAA. These findings align with the presence of genes in the C3^T^ genome associated with these PGP traits (Tables S1, S2 and S3). Similar characteristics have been reported in *Pantoea* species closely related to C3^T^, i.e. *Pantoea brenneri* [13], *P. agglomerans* [14] and *Pantoea eucalypti* [15], and in other species distantly related like *Pantoea alhagi* [65], *Pantoea phytobeneficialis* [53] and *Pantoea trifolii* [16].

The cellular fatty acid composition of strain C3^T^ was determined at Colección Española de Cultivos Tipo (Valencia, Spain) following the protocol recommended by the MIDI Microbial Identification System [66]. Cells were grown in nutrient agar at 30°C for 24 h, and the cellular fatty acid content was obtained using an Agilent 6850 gas chromatograph, with the MIDI Microbial Identification System employing the CLIN6 method [67].

Results showed that the predominant fatty acids of strain C3^T^ were C_16 : 0_ (31.6%); summed feature 3 containing C_16 : 1_ ω6c and/or C_16 : 1_ ω7c (24.6%); summed feature 8 containing C_18 : 1_ ω7c and/or C_18 : 1_ ω6c (15.2%); summed feature 2 containing C_12 : 0_ aldehyde and/or C_14 : 0_ 3OH and/or C_16 : 1_ iso I and/or an unidentified fatty acid with an equivalent chain length of 10.9525 (9.4%); and C_17  :  0_ cyclo (8.2%). While showing minor variations, these major compounds are consistent with those identified in other *Pantoea* species [68] (Table S6).

Based on genomic, phylogenetic, biochemical and chemotaxonomic evidence, we suggest that strain C3^T^ constitutes a new species within the genus *Pantoea*, for which we propose the name *P. phytostimulans* sp. nov.

## Description of *Pantoea phytostimulans* sp. nov.

*Pantoea phytostimulans* (phy.to.sti’mu.lans. Gr. neut. n. *phyton*, plant; L. pres. part. *stimulans*, stimulating; N.L. part. adj. *phytostimulans*, referring to its ability to promote plant growth). Cells are Gram-stain-negative, facultatively anaerobic, motile short rods (~0.8–1.2×1.6–2.6 µm) with a polar flagellum. Colonies on nutrient agar are yellowish, circular, moist and convex with 1.0–2.5 mm in diameter after 72 h at 30°C. Growth occurs at 4–36 °C (optimum, 28–30 °C), at a pH range of 5–9 (optimum, 6.5) and in the presence of 0–7% (w/v) NaCl (optimum, 1%). Catalase activity, NO_2_ production and *β*-galactosidase activity are positive, whereas oxidase activity, lysine and ornithine decarboxylase, tryptophan deaminase, urease, citrate utilization, production of H_2_S and indole are negative. Acids are produced from l-arabinose, d-ribose, d-xylose, d-galactose, d-glucose, d-fructose, d-mannose, l-rhamnose, inositol, d-mannitol, *N*-acetylglucosamine, arbutin, d-cellobiose, d-maltose, d-trehalose and 5-keto-gluconate; weakly produced from glycerol, salicin, d-melibiose, d-lyxose, d-fucose, gluconate and 2-keto-gluconate; and are not produced from erythritol, d-arabinose, l-xylose, d-adonitol, methyl-*β*-d-xylopyranoside, l-sorbose, dulcitol, d-sorbitol, methyl *α*-d-mannopyranoside, methyl *α*-d-glucopyranoside, amygdalin, aesculin, d-lactose, sucrose, inulin, d-melezitose, d-raffinose, starch, glycogen, xylitol, gentiobiose, d-turanose, d-tagatose, l-fucose, d-arabitol and l-arabitol. The predominant fatty acids are C_16 : 0_; summed feature 3 containing C_16 : 1_ ω6c and/or C_16 : 1_ ω7c; summed feature 8 containing C_18 : 1_ ω7c and/or C_18 : 1_ ω6c; summed feature 2 containing C_12 : 0_ aldehyde and/or C_14 : 0_ 3OH and/or C_16 : 1_ iso I and/or an unidentified fatty acid with an equivalent chain length of 10.9525; and C_17  :  0_ cyclo.

The type strain, C3^T^ (=CECT 31163^T^=NCIMB 15596^T^), was isolated from the rhizosphere of sorghum in Jumilla, Murcia, Spain. The genome size of the type strain is 5,058,178 bp, containing a chromosome of 3,989,142 bp, three megaplasmids and one short plasmid. The DNA G+C content is 51.5 mol% (calculated from the whole genome sequence). The GenBank accession numbers for the 16S rRNA partial gene and genome sequences of strain C3^T^ are PQ300029 and CP173698-CP173702, respectively.

## Supplementary material

10.1099/ijsem.0.006764 Uncited Supplementary Material 1.

## Abbreviations

ACTI: Área Científica y Técnica de Investigación
ANI: average nucleotide identity
BGCs: biosynthetic gene clusters
dDDH: digital DNA–DNA hybrization
GABA: gamma-aminobutyric acid
IAA: indole-3-acetic acid
LPSN: List of Prokaryotic Names with Standing in Nomenclature
ML: maximum likelihood
NBRIP: National Botanical Research Institute’s phosphate growth medium
NJ: neighbour-joining
PGP: plant growth-promoting
TYGS: Type (Strain) Genome Server
UMU: University of Murcia
