# Supplementary material for: Pantoea phytostimulans sp. nov., a novel strain with plant growth-promoting properties
Source: Int J Syst Evol Microbiol. 2025 Apr 25;75(4):006764. doi: 10.1099/ijsem.0.006764 (PMC12038178; doi:10.1099/ijsem.0.006764)
Supplement: Uncited Supplementary Material 1. [file ijsem-75-06764-s001.pdf]

Supplementary material.

Pantoea phytostimulans sp. nov., a novel strain with plant growth-promoting properties

Jonatan C. Campillo-Brocal, María Sánchez-García, Jorge Malo-López-Román, Antonio Bernal-Soro, Antonio Sánchez-Amat

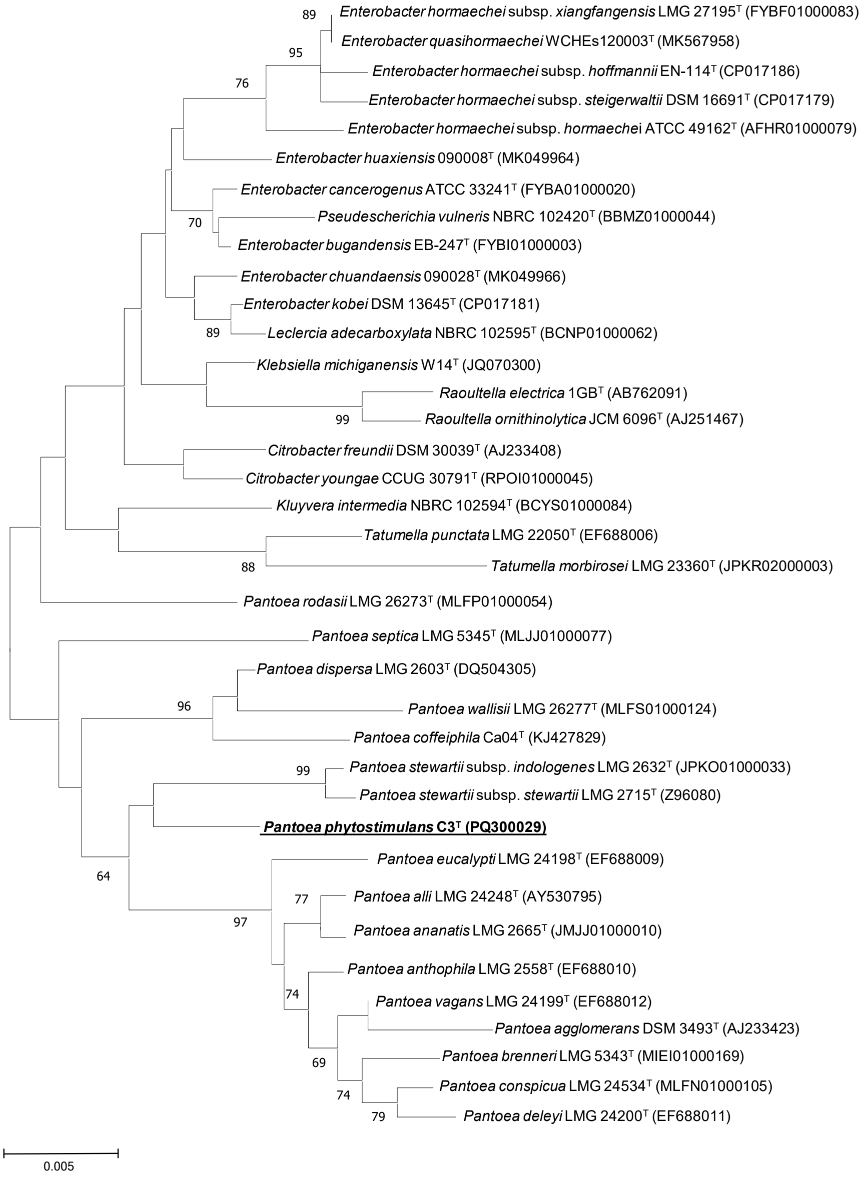

Fig. S1. Unrooted neighbor-joining tree based on 16S rRNA sequences illustrating the closest phylogenetic relatives of *Pantoea phytostimulans* sp. nov. C3^T. Bootstrap values, shown as percentages of 1000 replicates, are indicated at the branching points when >60%. Database accession numbers are provided in parentheses. Bar, 0.005 substitutions per nucleotide position.

Company Limited by Guarantee l Registered in England No. 1039582 l Registered Office Charles Darwin House, 12 Roger Street, WC1N 2JU, UK
Registered as a Charity: 264017 (England & Wales); SC039250 (Scotland)

(a) (b) (c) (d)

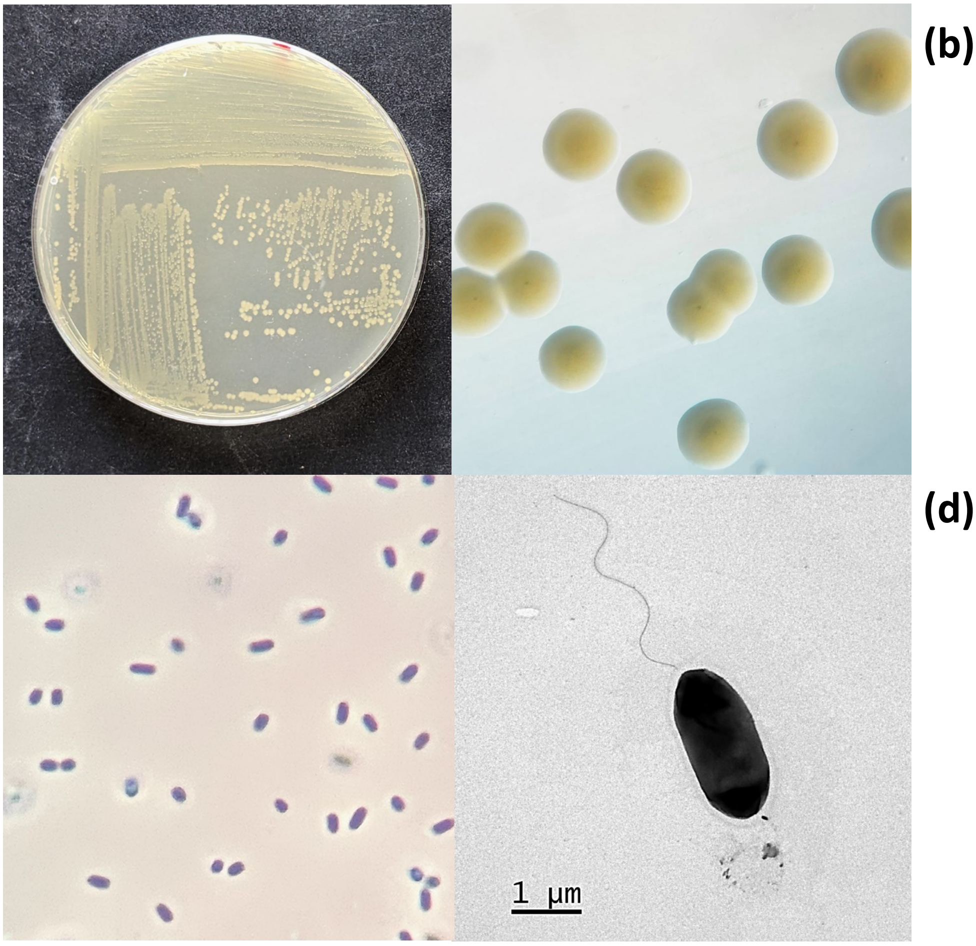

Fig. S2. Morphological characteristics of strain C3^T in nutrient medium. **(a)** Colony morphology on plates. **(b)** Colony morphology at higher amplification. **(c)** Cell morphology by phase contrast microscopy (1000x) and **(d)** by transmission electron microscopy stained with uranyl acetate showing the polar flagellum (8000x).

Table S1. Genes involved in phosphate solubilization and transport in strain C3^T.

Locus tag (GenBank accession)	Gene	Product	Pathway
ACF2G4_04860 (XLQ81641)	*pqqA*	pyrroloquinoline quinone precursor peptide	PQQ synthesis
ACF2G4_04865 (XLQ80782)	*pqqB*	pyrroloquinoline-quinone synthase	
ACF2G4_04870 (XLQ80783)	*pqqC*	pyrroloquinoline-quinone synthase	
ACF2G4_04875 (XLQ81642)	*pqqD*	pyrroloquinoline quinone biosynthesis peptide chaperone	
ACF2G4_04880 (XLQ80784)	*pqqE*	pyrroloquinoline quinone biosynthesis protein E	
ACF2G4_04885 (XLQ80785)	*pqqF*	pyrroloquinoline quinone biosynthesis protein F	

ACF2G4_16165 (XLQ79481)	*gcd*	Glucose dehydrogenase pyrroloquinoline quinone-dependent	D-gluconate production
ACF2G4_05045 (XLQ80816)	*gcd*		
ACF2G4_05800 (XLQ80958)	*gcd*		
ACF2G4_16900 (XLQ79616) ACF2G4_16110 (XLQ79471)	*gad*	Gluconate 2-dehydrogenase, membrane-bound, flavoprotein	2-ketogluconate production
ACF2G4_16905 (XLQ79617) ACF2G4_16105 (XLQ79470)	*gad*	Gluconate 2-dehydrogenase, membrane-bound, gamma subunit	
ACF2G4_16910 (XLQ79618) ACF2G4_16115 (XLQ79472)	*gad*	Gluconate 2-dehydrogenase, membrane-bound, cytochrome c	
ACF2G4_09550 (XLQ78270)	*ppx*	Exopolyphosphatase	Inorganic polyphosphates degradation
ACF2G4_03545 (XLQ80544)			
ACF2G4_11955 (XLQ78717)	*ppa*	Inorganic pyrophosphatase	
ACF2G4_06855 (XLQ81153)	*phoA*	Alkaline phosphatase	Organic phosphate solubilization
ACF2G4_20700 (XLQ82340)	*appA*	Phytase	
ACF2G4_02805 (XLQ80405)	*agp*	Glucose-1-phosphatase	
ACF2G4_07330 (XLQ81240) ACF2G4_07335 (XLQ81241)	*phnE*	Phosphonate transport system permease protein	Phosphonates transport and degradation
ACF2G4_07340 (XLQ81242)	*phnD*	Phosphonate transport system substrate-binding protein	
ACF2G4_07345 (XLQ81243)	*phnC*	Phosphonate transport system ATP-binding protein	
ACF2G4_07350 (XLQ81244)	*phnP*	Phosphonate metabolism protein	
ACF2G4_07355 (XLQ81245)	*phnN*	Ribose 1,5-bisphosphate phosphokinase	
ACF2G4_07360 (XLQ81246)	*phnM*	Alpha-D-ribose 1-methylphosphonate 5-triphosphate diphosphatase	
ACF2G4_07365 (XLQ81247)	*phnL*	Phosphonate C-P lyase system protein	
ACF2G4_07370 (XLQ81248)	*phnK*		
ACF2G4_07375 (XLQ81249)	*phnJ*	Carbon-phosphorus lyase complex subunit	
ACF2G4_07380 (XLQ81250)	*phnI*		
ACF2G4_07385 (XLQ81251)	*phnH*	Phosphonate C-P lyase system protein	
ACF2G4_07390 (XLQ81252)	*phnG*		

ACF2G4_07395 (XLQ81253)	*phnF*	Phosphonate metabolism transcriptional regulator	
ACF2G4_09530 (XLQ78266) ACF2G4_14475 (XLQ79182)	*pstB*	Phosphate transport system ATP-binding protein	Phosphate transport
ACF2G4_09535 (XLQ78267) ACF2G4_14470 (XLQ79181)	*pstA*	Phosphate transport system permease protein	
ACF2G4_14460 (XLQ79179)	*pstS*	Phosphate transport system substrate-binding protein	
ACF2G4_14465 (XLQ79180)	*pstC*	Phosphate transport system permease protein	
ACF2G4_14480 (XLQ79183)	*phoU*	Phosphate transport system regulatory protein	

Table S2. Genes involved in siderophore biogenesis and transport in strain C3^{T}.

**Locus tag (GenBank accession)**	**Gene**	**Product**	**Pathway**
ACF2G4_16535 (XLQ79551)	*entH*	Thioesterase	Enterobactin synthesis
ACF2G4_16540 (XLQ79552)	*entA*	2,3-dihydro-2,3-dihydroxybenzoate dehydrogenase	
ACF2G4_16545 (XLQ79553)	*entB*	Isochorismatase	
ACF2G4_16550 (XLQ79554)	*entE*	2,3-dihydroxybenzoate-AMP ligase	
ACF2G4_16555 (XLQ79555)	*entC*	Isochorismate synthase	
ACF2G4_16585 (XLQ79561)	*entF*	Enterobactin synthase component F	
ACF2G4_16590 (XLQ79562)	*mbtH*	MbtH family protein	
ACF2G4_16595 (XLQ81737)	*fes*	Enterochelin esterase	
ACF2G4_16560 (XLQ79556)	*fepB*	Ferric enterobactin ABC transporter substrate-binding protein	Enterobactin transport
ACF2G4_16565 (XLQ79557)	*entS*	Enterobactin exporter	
ACF2G4_16570 (XLQ79558)	*fepD*	Ferric enterobactin ABC transporter membrane subunit	
ACF2G4_16575 (XLQ79559)	*fepG*		
ACF2G4_16580 (XLQ79560)	*fepC*	Ferric enterobactin ABC transporter ATP binding subunit	
ACF2G4_16600 (XLQ79563)	*fepA*	TonB-dependent siderophore receptor	

ACF2G4_18065 (XLQ79832)	*fhuA*	Ferric hydroxamate outer membrane receptor	Hydroxamate siderophores transport
ACF2G4_18070 (XLQ79833)	*fhuC*	Ferric hydroxamate ABC transporter ATP-binding protein	
ACF2G4_18075 (XLQ79834)	*fhuD*	Ferric hydroxamate ABC transporter substrate-binding protein	
ACF2G4_18080 (XLQ79835)	*fhuB*	Ferric hydroxamate ABC transporter permease	

Table S3. Genes involved in indole-3-acetic acid (IAA), cytokinin and gamma-aminobutyric acid (GABA) production in strain C3^T.

Locus tag (GenBank accession)	Gene	Product	Pathway
ACF2G4_05950 (XLQ80988)	*trpH*	PHP domain-containing anthranilate phosphoribosyltransferase	L-tryptophan synthesis (IAA production)
ACF2G4_05955 (XLQ80989)	*trpE*	Anthranilate synthase component 1	
ACF2G4_05960	*trpD*	Anthranilate phosphoribosyltransferase TrpD	
ACF2G4_05965 (XLQ80990)	*trpCF*	Bifunctional indole-3-glycerol-phosphate synthase TrpC	
ACF2G4_05970 (XLQ80991)	*trpB*	Tryptophan synthase subunit beta	
ACF2G4_05975 (XLQ80992)	*trpA*	Tryptophan synthase subunit alpha	
ACF2G4_12790 (XLQ78871)	*trpS*	Tryptophanyl-tRNA synthetase	
ACF2G4_17395 (XLQ79705)	*trpR*	Trp operon repressor	
ACF2G4_09090 (XLQ81562)	*ipdC*	Indolepyruvate decarboxylase	IAA production (IPyA pathway)
ACF2G4_07505 (XLQ81272)	*aldH*	Aldehyde dehydrogenase	
ACF2G4_12105 (XLQ78746)	*miaA*	tRNA dimethylallyltransferase	Cytokinin synthesis and transformation
ACF2G4_01420 (XLQ80160)	*miaB*	tRNA-2-methylthio-N6-dimethylallyladenosine synthase	
ACF2G4_05280 (XLQ80858)	*xdhC*	Xanthine dehydrogenase accessory protein XdhC	
ACF2G4_05285 (XLQ80859)	*xdhB*	Xanthine dehydrogenase molybdopterin binding subunit	
ACF2G4_05290 (XLQ80860)	*xdhA*	Xanthine dehydrogenase small subunit	
ACF2G4_07690 (XLQ81303)	*puuD*	Gamma-glutamyl-gamma-aminobutyrate hydrolase	GABA synthesis and degradation
ACF2G4_07695 (XLQ81304)	*puuR*	HTH-type transcriptional regulator PuuR	
ACF2G4_08585 (XLQ81472)	*puuE*	4-aminobutyrate aminotransferase	
ACF2G4_07450 (XLQ81262)	*gabT*		
ACF2G4_02810 (XLQ80406)	*gabD*	succinate-semialdehyde dehydrogenase / glutarate-semialdehyde dehydrogenase	
ACF2G4_07510 (XLQ81273)			

Table S4. The biosynthetic gene clusters (BGCs) identified by antiSMASH in strain C3^T.

Type	Similarity	Most similar known cluster	Size (kb)	BGC location
NRP-metallophore	100%	Enterobactin	53.8	Chromosome: 3,522,175 - 3,575,974
Arylpolyene, hserlactone	94%	Aryl polyene	61.2	Chromosome: 605,628 - 666,799
Terpene	100%	Carotenoid	25.8	pC3_1: 220,235 - 246,011
NRP-metallophore	16%	Photobactin	44.5	pC3_2: 193,592 - 238,061
Thiopeptide	14%	O-antigen	26.3	Chromosome: 512,262 - 538,526
Redox-cofactor	-	-	22.2	Chromosome: 1,010,625 - 1,032,791

Table S5. API 10 S and API 50 CH test results for *Pantoea phytostimulans* C3^T.

**API 10 S**			
**Characteristics**	**24 h**	**Characteristics**	**24 h**
D-Glucose fermentation	+	Beta-galactosidase	+
L-Arabinose fermentation	+	Urease	-
Citrate utilization	w	Lysine decarboxylase	-
H_2S production	-	Ornithine decarboxylase	-
Indole production	-	Tryptophan deaminase	-
NO_2 production	+	Cytochrome oxidase	-

**API 50 CH**					
**Characteristics**	**24 h**	**48 h**	**Characteristics**	**24 h**	**48 h**
050CHB/E medium control	–	–	Esculin ferric citrate	-	-
Glycerol	w	w	Salicin	-	w
Erythritol	-	-	D-Cellobiose	+	+
D-Arabinose	-	-	D-Maltose	+	+
L-Arabinose	+	+	D-Lactose	-	-
D-Ribose	+	+	D-Melibiose	-	w
D-Xylose	+	+	Sucrose	-	-
L-Xylose	-	-	D-Trehalose	+	+
D-Adonitol	-	-	Inulin	-	-
Methyl-β-D-xylopyranoside	-	-	D-Melezitose	-	-
D-Galactose	+	+	D-Raffinose	-	-
D-Glucose	+	+	Starch	-	-
D-Fructose	+	+	Glycogen	-	-
D-Mannose	+	+	Xylitol	-	-
L-Sorbose	-	-	Gentiobiose	-	-
L-Rhamnose	+	+	D-Turanose	-	-
Dulcitol	-	-	D-Lyxose	w	w

Inositol	+	+	D-Tagatose	-	-
D-Mannitol	+	+	D-Fucose	-	w
D-Sorbitol	-	-	L-Fucose	-	-
Methyl-α-D-mannopyranoside	-	-	D-Arabitol	-	-
Methyl-α-D-glucopyranoside	-	-	L-Arabitol	-	-
N-acetyl-glucosamine	+	+	Gluconate, K	w	w
Amygdalin	-	-	2-Ketogluconate, K	w	w
Arbutin	+	+	5-Ketogluconate, K	w	+

According to the API manual, test results for API 10 S were recorded at 30 °C for 24 h, and for both 24 h and 48 h for API 50 CH. +, positive; w, weak positive; −, negative.

Table S6. Relative cellular fatty acid content (%) of strain C3^T and closest phylogenetic strains with available data.

Cellular fatty acid	***Pantoea phytostimulans*** **C3^T**	***Pantoea agglomerans*** **NBRC 102470^T**	***Pantoea vagans*** **LMG 24199^T**	***Pantoea. rwandensis*** **LMG 26275^T**	***Pantoea ananatis*** **LMG 2676**
$C_{12:0}$	3.8	3.8	6.4	4.2	11.0
$C_{14:0}$	5.7	6	7.3	6.9	5.5
$C_{15:0}$	-	1.1	-	-	-
$C_{15:0}$ 3-OH	-	13.2	-	-	-
$C_{16:0}$	31.6	27.1	25	26.1	18.0
$C_{16:1}$	-	17.2-26.7	-	-	-
$C_{17:0}$	0.7	-	-	-	-
$C_{17:0}$ cyclo	8.2	13.2	7.2	7.1	1.9
$C_{18:0}$	0.4	-	-	-	-
$C_{19:0}$ cyclo ω8*c*	0.4	-	-	-	-
Summed feature 2	9.4	17.2⁻26.7	-	14.3	-
Summed feature 3	24.6	-	32.0	-	22.0
Summed feature 8	15.2	-	9.3	11.8	10.0

All data retrieved from (1), except data for *Pantoea phytostimulans* C3^T (this study). Summed features refer to combinations of fatty acids that the MIDI system cannot distinguish individually. Summed feature 2 includes a mixture of $C_{12:0}$ aldehyde and/or $C_{14:0}$ 3OH and/or $C_{16:1}$ iso I, and/or an unidentified fatty acid with an equivalent chain length of 10.9525. Summed feature 3 consists of $C_{16:1}$ω7c and/or $C_{16:1}$ω6c, while summed feature 8 includes $C_{18:1}$ω7c and/or $C_{18:1}$ω6c.

References

1. **Biswas R, Misra A, Ghosh S, Chakraborty A, Mukherjee P, Dam B**. *Pantoea tagorei* sp. nov., a rhizospheric bacteria with plant growth-promoting activities. *Indian J Microbiol*. 2024;64(3):937-49
